# Characteristics of Carbapenemase-Producing *Klebsiella pneumoniae* Isolated in the Intensive Care Unit of the Largest Tertiary Hospital in Bangladesh

**DOI:** 10.3389/fmicb.2020.612020

**Published:** 2021-01-15

**Authors:** Takashi Okanda, Anwarul Haque, Takuro Koshikawa, Amirul Islam, Qumrul Huda, Hiromu Takemura, Tetsuya Matsumoto, Shigeki Nakamura

**Affiliations:** ^1^Department of Microbiology, Tokyo Medical University, Tokyo, Japan; ^2^Department of Microbiology, St. Marianna University School of Medicine, Kawasaki, Japan; ^3^Department of Infectious Diseases, International University of Health and Welfare, Narita, Japan; ^4^Department of Anesthesia, Analgesia and Intensive Care Medicine, Bangabandhu Sheikh Mujib Medical University, Dhaka, Bangladesh

**Keywords:** PDR, ST147, NDM-1, NDM-5, OXA-181, OXA-232

## Abstract

For addressing the issue of antimicrobial drug resistance in developing countries, it is important to investigate the characteristics of carbapenemase-producing organisms. We aimed to genetically characterize a carbapenemase-producing *Klebsiella pneumoniae* (CPKP) isolated in the intensive care unit of a tertiary hospital in Bangladesh. The number of CPKP isolates were 43/145 (30%), of which pandrug-resistant (PDR) strains were 14%. These carbapenemases were New Delhi metallo-beta-lactamase (NDM)-1 (53%), NDM-5 (14%), oxacillinase (OXA)-181 (12%), OXA-232 (10%), NDM-5 + OXA-181 (5%), and NDM-5 + OXA-232 (2%). Many CPKP isolates harbored a variety of resistance genes, and the prevalence of 16S rRNA methyltransferase was particularly high (91%). The 43 CPKP isolates were classified into 14 different sequence types (STs), and the common STs were ST34 (26%), ST147 (16%), ST11 (9%), ST14 (9%), ST25 (7%), and ST231 (7%). In this study, PDR strains were of three types, ST147, ST231, and ST14, and their PDR rates were 57, 33, and 25%, respectively. The spread of the antimicrobial drug resistance of CPKP in Bangladesh was identified. In particular, the emergence of PDR is problem, and there may be its spread as a superbug of antimicrobial treatment.

## Introduction

The worldwide spread of carbapenemase-producing organisms (CPOs) is increasing (Bonomo et al., 2009). Carbapenem resistance in *Klebsiella pneumoniae* is frequently associated with antimicrobial resistance to other antibiotic classes, making treatment of infections challenging (Fournier et al., 2018). In North America, South America, and Europe, the main carbapenemase-producing genes are the *Klebsiella pneumoniae* carbapenemase (KPC) types; in the Middle East and Africa, they are the oxacillinase-48-like (OXA-48-like) types; and in the Indian subcontinent, they are of the New Delhi metallo-beta-lactamase (NDM) and OXA-48-like types. Many of these multidrug-resistant (MDR) strains are sequence type (ST) 11, ST14, ST37, ST147, and ST258 and are spreading worldwide as international MDR high-risk clones (Mathers et al., 2015).

The Center for Disease Control and Prevention (CDC) and the European Centre for Disease Prevention and Control have jointly developed definitions for MDR, extensively drug-resistant (XDR), and pandrug-resistant (PDR) bacteria (Magiorakos et al., 2012). *K. pneumoniae* strains classified as XDR are rapidly emerging due to the dissemination of resistance toward aminoglycosides, β-lactams, fluoroquinolones, and carbapenems (Al-Marzooq et al., 2015). Recently, XDR strains have evolved to become PDR by acquiring resistance to tigecycline and polymyxin antibiotics (Papadimitriou-Olivgeris et al., 2018). The spread of such strains is associated with high mortality rates, limited treatment options, and rapid dissemination of successful bacterial clones in the hospital setting (Guo et al., 2016; Protonotariou et al., 2018).

Antimicrobial resistance (AMR) is a major problem in Asia (O’neill, 2016). Bangladesh is a developing country surrounded by India and is known to have a high prevalence of carbapenem-resistant organisms. The CPOs detected are mainly NDM-producing strains, but in recent years, OXA-48-like-producing strains have also been detected. However, no surveillance system has been established in developing countries in this region, and there are few reports on molecular epidemiology (Hasan and Rabbani, 2019). We started the short-term prospective study of MDR bacteria with the ultimate goal of building a resistant surveillance system in Bangladesh (Okanda et al., 2018). In this study, we performed an analysis of acquired drug resistance mechanism and molecular epidemiology of carbapenemase-producing *Klebsiella pneumoniae* (CPKP) isolated in a tertiary hospital in Dhaka, Bangladesh.

## Materials and Methods

### Collection of Isolates

Bangabandhu Sheikh Mujib Medical University Hospital is the premier Postgraduate Medical Institution of Bangladesh and a major tertiary hospital with 1,900 beds. The intensive care unit (ICU) at this hospital accepts critically ill patients not only throughout Dhaka but also from neighboring countries. In this study, collection of isolates was originally scheduled for only between August and October 2015, but because of observed subsequent changes in AMR at ICU, isolates were also collected between August and September 2017. A total of 145 third-generation cephalosporin-resistant *Klebsiella pneumoniae* were isolated from blood, sputum or tracheal aspirate, urine, and pus in ICU patients (82 in 2015 and 63 in 2017). These were all consecutive isolates selected on the CHROMagar ESBL medium (Kanto Chemical Co., Inc., Japan), eliminating duplication by patient. These isolates were inoculated into casitone medium and transported to Tokyo Medical University in Japan, where they were incubated on modified Drigalski agar (EIKEN Chemical Co., Ltd) at 35°C for 18 h. The resulting cultures were re-identified by matrix-assisted laser desorption/ionization time-of-flight mass spectrometry using MALDI Biotyper (library ver. 9.0.0.0) and stored at −80°C in heart infusion broth containing 20% glycerol until use.

### Screening of Carbapenemase-Producer and Detection of Carbapenemase-Encoding Genes

Phenotypic detection of carbapenemase was performed using the modified carbapenem inactivation method (mCIM) and Carba NP test. *Escherichia coli* ATCC 25922 was used as an indicator organism and negative control, whereas *K. pneumoniae* KPP 127 was used as a positive control. All isolates that showed positive results and intermediate results were detected of carbapenemase-encoding genes by polymerase chain reaction (PCR) assay. DNA was extracted using the Cica Geneus DNA Extraction Reagent (Kanto Chemical Co., Inc., Japan). The examined carbapenemase-encoding genes were *bla*_*KPC*_, *bla*_*GES*_, *bla*_*IMP*_, *bla*_*VIM*_, *bla*_*NDM*_, *bla*_*OXA–*__48__–like_, *bla*_*BIC*_, *bla*_*AIM*_, *bla*_*GIM*_, *bla*_*SIM*_, *bla*_*DIM*_, and *bla*_*SPM*_ (Supplementary Table S1).

### Genotypic Detection of Carbapenemase-Encoding Genes and Other Resistance Genes

The confirmed CPKP isolates were tested for extended-spectrum β-lactamases (ESBLs)-producing genes, plasmid-mediated AmpC β-lactamase (PABLs)-producing genes, mobilized colistin resistance (*mcr*) gene, 16S rRNA methyltransferase (16SRMTase) genes, tetracycline resistance genes, plasmid-mediated quinolone resistance (PMQR) genes, fosfomycin resistance genes, sulfonamide resistance genes, and dihydrofolate reductase (DHFR)-encoding genes (Supplementary Table S1). Target genes were confirmed via single PCR, and PCR fragments were then purified from PCR product or agarose gels using Cica Geneus^®^ PCR & Gel Prep Kit (Kanto Chemical Co., Inc., Japan). Following sequencing, the similarity search was performed with the sequenced data using BLAST analysis^1^.

### Antimicrobial Susceptibility Testing

Carbapenemase-producing *Klebsiella pneumoniae* isolates were tested for their susceptibility to piperacillin/tazobactam (TZP), cefepime (FEP), cefmetazole (CMZ), moxalactam (MOX), flomoxef (FOX), aztreonam (ATM), imipenem (IPM), meropenem (MEM), doripenem (DOR), biapenem (BPM), colistin (CST), gentamicin (GEN), amikacin (AMK), minocycline (MIN), tigecycline (TGC), ciprofloxacin (CIP), levofloxacin (LVX), fosfomycin (FOF), and trimethoprim/sulfamethoxazole (SXT). Microdilution susceptibility testing was performed and minimum inhibitory concentrations (MIC) determined according to the Clinical and Laboratory Standards Institute (CLSI) guidelines (Clinical and Laboratory Standards Institute (CLSI), 2020). In this edition, CST breakpoints were defined as resistant ≥4 mg/l and intermediate ≤2 mg/l, with no susceptible category defined. In addition, no TGC and FOF breakpoints were defined for *K. pneumoniae*. Thus, CST, TGC, and FOF breakpoints were performed according to the European Committee on Antimicrobial Susceptibility Testing (EUCAST) guideline^2^. *E. coli* ATCC 25922 was used for quality control of the test.

### Molecular Epidemiology Typing

Pulsed-field gel electrophoresis (PFGE) was carried out by the following method. After digestion of the genomic DNA by *Xba*I (New England Biolabs), the restriction fragments were separated using a temperature-controlled CHEF-DR III system (Bio-Rad) for 19.5 h at 14°C under the following conditions: two state modes, initial switch time of 5.3 s, final switch time of 60.0 s, a gradient of 6 V/cm, and switch angle of 120°. After staining with ethidium bromide, the fragments were visualized using an ultraviolet transilluminator. Percent similarities were determined by generating a dendrogram by the unweighted pair group method using arithmetic averages and based on Dice coefficients. Band position tolerance and optimization were both set at 1.0%. A similarity coefficient of 80.0% was selected to define the clusters after reviewing the epidemiological data associated with each isolate cluster. For the determination of PCR conditions and primer sequences, information obtained from the Institut Pasteur multilocus sequence typing (MLST) database^3^ was used (Diancourt et al., 2009). For the identification of ST, the allelic profiles of the purified sequences were submitted to the Institut Pasteur MLST database. Clustering of each ST to determine the clonal group (CG) of single-locus mutants was conducted using the eBURST program (Feil et al., 2004).

## Results

### CPKP Isolates

**FIGURE 1:**
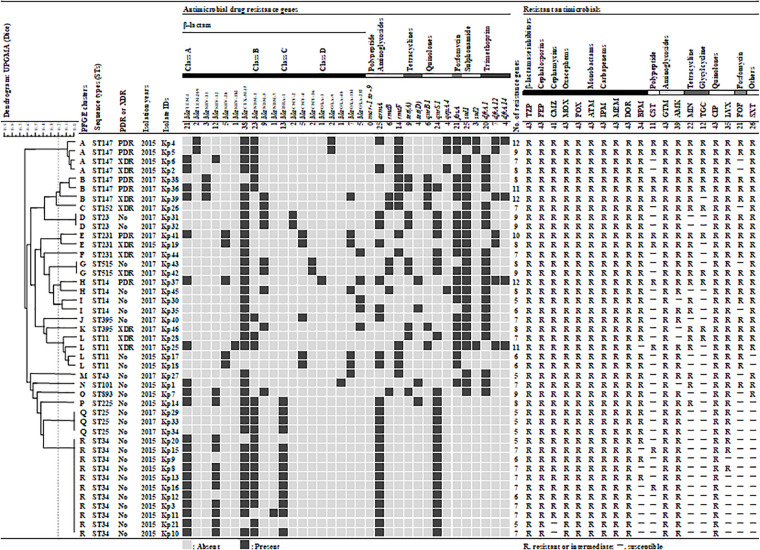
Genetic and antibiogram profiles of 43 carbapenemase-producing *Klebsiella pneumoniae* isolates. A similarity coefficient of 80% was selected to define the clusters after reviewing the epidemiological data associated with each of the clusters of isolates. The presence or absence of plasmid-mediated antimicrobial resistance genes is indicated by black filled square or gray filled square, respectively. Antimicrobial susceptibility: susceptible (S) and resistant/intermediate (–). TZP, piperacillin/tazobactam; FEP, cefepime; CMZ, cefmetazole; MOX, moxalactam; FOX, flomoxef; ATM, aztreonam; IPM, imipenem; MEM, meropenem; DOR, doripenem; BPM, biapenem; CST, colistin; GEN, gentamicin; AMK, amikacin; MIN, minocycline; TGC, tigecycline; CIP, ciprofloxacin; LVX, levofloxacin; FOF, fosfomycin; SXT, trimethoprim/sulfamethoxazole.

By phenotypic testing and sequencing, carbapenemase-producing genes were detected from 43 of the 145 isolates (30%). These 43 carbapenemase-producing genes were identified as *bla*_*NDM–*__1_ (23, 53%), *bla*_*NDM–*__5_ (6, 14%), *bla*_*OXA–*__181_ (5, 12%), *bla*_*OXA–*__232_ (4, 10%), 2 *bla*_*NDM–*__5_ + *bla*_*OXA–*__181_ (2, 5%), *bla*_*NDM–*__5_ + *bla*_*OXA–*__232_ (1, 2%), *bla*_*NDM–*__7_ (1, 2%), and *bla*_*OXA–*__48_ (1, 2%) by sequence. Therefore, 40 (93%) of the 43 CPKP isolates were single-harboring *bla*_*NDM*_ (30, 70%) or *bla*_*OXA–*__48__–like_ (10, 23%), while the remaining 3 (7%) were co-harboring both genes. CPKP isolates were co-harboring various AMR genes (Figure 1). ESBL- and PABL-encoding genes were detected in 43 (100%) and 22 (51%) CPKP isolates, respectively. Therefore, 22 CPKP isolates (51%) were co-harboring ESBLs and PABLs. In addition, the prevalence of 16SRMTase, tetracycline resistance, PMQR, fosfomycin resistance, sulfonamide resistance, and DHFR-encoding genes in CPKP isolates were 91% (39), 30% (13), 77% (33), 49% (21), 60% (26), and 60% (26), respectively. However, the *mcr* gene reported to date was not detected from colistin-resistant isolates.

### Antimicrobial Resistance

The susceptibility of 43 CPKP isolates are shown in Table 1. Forty-three CPKP isolates were resistant to many of the antibiotics tested, with 10 isolates (23%) classified as XDR and 6 isolates (14%) classified as PDR. Interestingly, these CPKP isolates were highly resistant to aminoglycosides, and all AMK-resistant isolates showed MIC ≥ 512. From 2015 to 2017, the resistant rates of BPM, MIN, TGC, LVX, FOF, and SXT were increased, while the CST susceptibility was almost unchanged. In addition, the MIC value of six PDR isolates are shown in Table 2. PDR isolates showed high-level resistance to many antimicrobials, but low-level resistance to CST and TGC.

**TABLE 1:** Susceptibility of carbapenemase-producing *Klebsiella pneumoniae* in Bangladesh.

	2015	2017	Total
Number of isolates	21/82 (26%)	22/63 (35%)	43/145 (30%)
**Antimicrobial resistance types**			
Multidrug resistant	16/21 (76%)	11/22 (50%)	27/43 (63%)
Extensively drug resistant	3/21 (14%)	7/22 (32%)	10/43 (23%)
Pandrug resistant	2/21 (10%)	4/22 (18%)	6/43 (14%)
**Resistant rate**			
Piperacillin–tazobactam	100%	100%	100%
Cefepime	100%	100%	100%
Cefmetazole	90%	100%	95%
Moxalactam	100%	100%	100%
Flomoxef	100%	100%	100%
Aztreonam	100%	100%	100%
Imipenem	100%	100%	100%
Meropenem	100%	100%	100%
Doripenem	100%	100%	100%
Biapenem	62%	95%	79%
Colistin	24%	27%	26%
Gentamicin	100%	100%	100%
Amikacin	95%	86%	91%
Minocycline	33%	68%	51%
Tigecycline	19%	36%	28%
Ciprofloxacin	100%	100%	100%
Levofloxacin	62%	100%	81%
Fosfomycin	29%	68%	49%
Trimethoprim–sulfamethoxazole	33%	86%	60%

*Breakpoints are performed according to the Clinical and Laboratory Standards Institute guideline M100-30 edition and according to the European Committee on Antimicrobial Susceptibility Testing guideline ver.10.0 for colistin, tigecycline, and fosfomycin.*

**TABLE 2:** Susceptibility of pandrug-resistant carbapenemase-producing *Klebsiella pneumoniae* in Bangladesh.

ID	Sequence Types (MLST)	Carbapene- mases	MIC (μ g/mL)																		
			TZP	FEP	CMZ	MOX	FOX	ATM	IPM	MEM	DOR	BPM	CST	GEN	AMK	MIN	TGC	CIP	LVX	FOF	SXT
Kp4	ST147	NDM-1	>64/4	>16	128	128	>32	>256	64	32	32	4	4	>8	>1024	16	2	>2	64	128	>2/38
Kp5	ST147	NDM-1	>64/4	>16	128	128	>32	>256	64	32	32	4	4	>8	>1024	16	2	>2	8	128	>2/38
Kp36	ST147	NDM-1	>64/4	>16	256	256	>32	64	16	64	64	4	4	>8	>1024	8	2	>2	64	128	>2/38
Kp37	ST147	OXA-232	>64/4	>16	>256	64	>32	256	8	16	16	8	4	>8	>1024	64	8	>2	128	128	>2/38
Kp38	ST14	NDM-1	>64/4	>16	256	256	>32	64	32	64	64	16	4	>8	>1024	32	2	>2	128	128	>2/38
Kp41	ST231	OXA-181	>64/4	>16	>256	64	>32	256	16	32	16	16	4	>8	>1024	16	1	>2	64	128	>2/38

**FIGURE 2:**
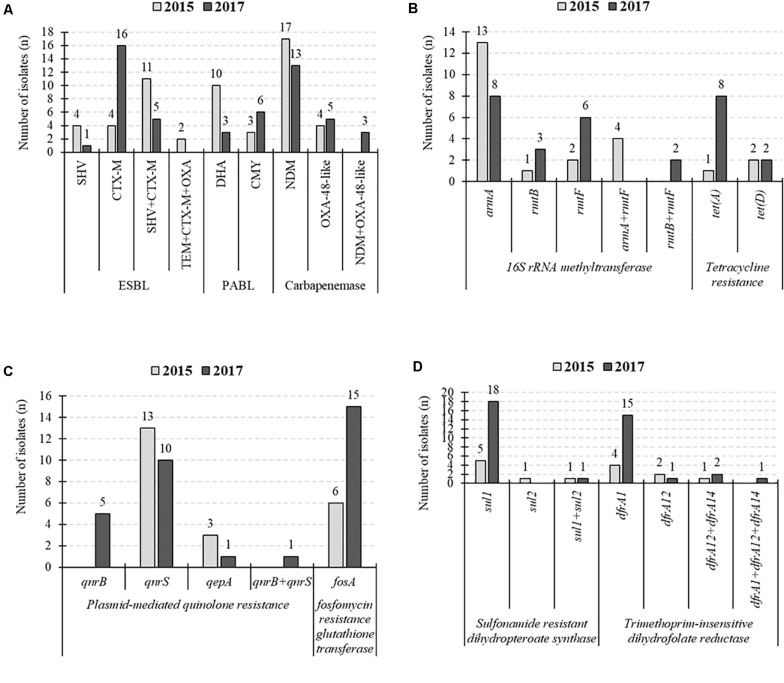
Changes in the number of detected resistance genes. **(A)** Extended-spectrum β-lactamases (ESBLs)-, plasmid-mediated AmpC β-lactamase (PABLs)-, and carbapenemase-producing genes. **(B)** 16S rRNA methyltransferase- and tetracycline resistance protein-producing genes. **(C)** Plasmid-mediated quinolone resistance and fosfomycin resistance genes. **(D)** Sulfonamide resistance genes and dihydrofolate reductase encoding genes.

CTX-M-15 was the common ESBL, but PABL was changed from DHA to CMY in 2017 (Figure 2). In 16SRMTase, the prevalence did not change during the collection period, while the common type was changed from the *armA* gene to the *rmtB/F* genes. The 2017 CPKP isolate showed an increased prevalence of some resistance genes compared to the 2015 isolate. In tetracycline resistance, the prevalence of the *tet (A)* gene increased from 5% (1/21) to 36% (8/22). Perhaps this is the cause of the increased MINO or TGC resistance rate. In addition, the prevalence of the *fosA*, *sul1*, and *dfrA1* genes were increased from 29% (6/21) to 68% (15/22), from 29% (6/21) to 86% (19/22), and from 19% (4/21) to 73% (16/22), respectively. As the number of these resistance genes increased, the resistance rate of the corresponding antibacterial drug increased. In fact, the increase in resistant rates from 2015 to 2017 was 29% (6/21) to 68% (15/22) for FOF and 33% (7/21) to 86% (19/22) for SXT.

### Molecular Epidemiological Characteristics of CPKP

Pulsed-field gel electrophoresis identified 29 different banding patterns from 43 CPKP isolates. These banding patterns were classified into 18 clusters (A to R) by more than 80% genetic associations (Figure 1). Common clusters were R (11, 26%), A (4, 9%), and L (4, 9%), and the most common cluster R was all ST34 strain. All ST34s were isolated from the ICU in 2015, and their antimicrobial susceptibility and acquisition resistance genes were almost the same. This suggested that there was an outbreak of ST34 strains in 2015. The STs of clusters A and L were ST147 and ST11, respectively. On the other hand, the second most common cluster A was all ST147, but it may be a different clone due to different banding patterns and separation dates. The band pattern of ST147 was significantly different between 2015 and 2017, forming cluster A and B, respectively. Therefore, it was considered that the transmission route was different between the ST147 strain in 2015 and in 2017.

**FIGURE 3:**
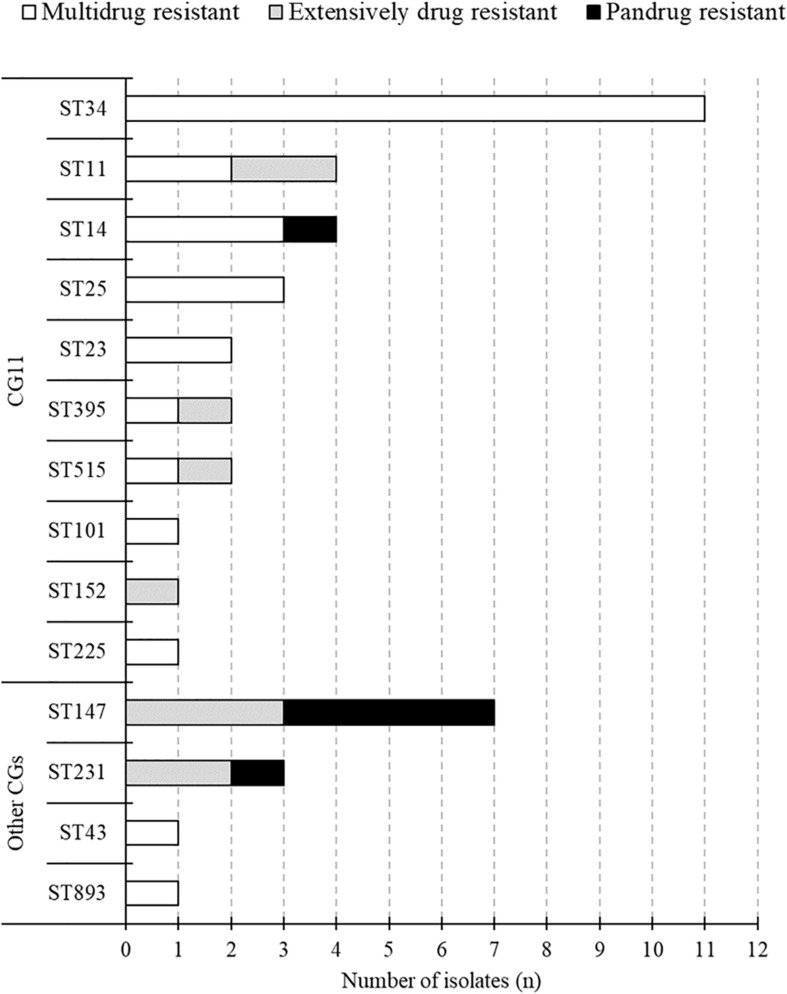
Classification of multidrug resistance type of each STs.

The MLST classified the 43 isolates into 14 different STs (Figure 1). The common STs were ST34 (11, 26%), ST147 (7, 16%), ST11 (4, 9%), ST14 (4, 9%), ST25 (3, 7%), and ST231 (3, 7%). ST147, ST11, and ST231 strains were isolated in both 2015 and 2017. The eBURST analysis, based on the MLST database, found that 14 different STs were clustered into four different CGs (Figure 3). The founder of each CG was ST11, ST147, ST231, and ST43, while ST893 did not form a cluster and was a singleton. CG11 was the most common group, but those STs were diverse, and only ST11 strain was isolated in both years. The multidrug resistance of ST147 and ST231 strains was serious, all of which were XDR or PDR.

## Discussion

The global spread of CPOs is a major issue in AMR measures, and the emergence of PDR strains makes antimicrobial treatment more difficult. The worsening of resistance of *K. pneumoniae* is remarkable, and some international MDR high-risk clones are reported (Mathers et al., 2015). These clones have a significant burden on global public health. Therefore, it is necessary to conduct intensive studies to monitor *K. pneumoniae* international MDR high-risk clones, to mitigate this issue. In the present study, we report for the molecular epidemiological characteristics of CPKP isolates collected at the largest tertiary hospital in Bangladesh. It is worth noting that PDR strains are ST147, ST231, and ST14, which harbored various resistance genes in addition to *bla*_*NDM*_- and *bla*_*OXA–*__48__–like_-encoding genes.

Antimicrobial resistance is globally increasing via the spread of carbapenemase, and treatment options are increasingly limited (Bonomo et al., 2009). In several cases, CPE are also resistant to colistin and tigecycline, and our data agrees with this observation (Table 2). In our study, the colistin resistance rate of CPKP isolates was 26% (MIC = 4 mg/l). Colistin resistance is caused by a decrease in negative charge due to modification of the lipid A structure of the outer membrane LPS. Modifications include those by the acquired *mcr* gene and those by the two-component regulatory system caused by chromosomal gene mutations (Macesic et al., 2020). However, the *mcr* gene was not detected from CST-resistant isolates. Thus, it is possible that the colistin resistance mechanism in this study is due to mutations in chromosomal resistance genes. Although the mechanism of tigecycline resistance has not been fully elucidated, the tigecycline resistance rate increased from 19% to 36% with the increase in the *tet(A)* gene. This result suggests that the tetracycline resistance protein is involved in tigecycline resistance of CPKP isolates. Resistance to CST and TGC has been on the rise because these antibiotics are regularly prescribed due to the scarcity of treatment options for CPE (5, 42–44). A report stating that 45% of 298 *K. pneumoniae* isolated at an Indian tertiary hospital between 2013 and 2017 were XDR and 9% were PDR is alarming (Mohapatra et al., 2018). Unfortunately, Mohapatra et al. (2018) do not mention the molecular epidemiology of XDR and PDR strains, but the possibility that these isolates are spreading from India and neighboring developing countries is plausible and worth investigating further.

Outbreaks involving the ST147 clone have been reported worldwide (Guo et al., 2016; Hamzaoui et al., 2018; Protonotariou et al., 2018). In Greece, the ST147 clone co-producing KPC-2 and VIM-1 was isolated from 25 patients at a tertiary teaching hospital, and 17 of these patients developed bloodstream infections (mortality rate = 48%) (Protonotariou et al., 2018). Nahid et al. (2017) provided insight into the emergence of ST147 PDR clone and the rapid global spreading of this high-risk clone. ST147 has been commonly reported among Indian isolates and has been frequently associated with NDM-1 and OXA48-like carbapenemases as observed from the present study (Pragasam et al., 2017). Indeed, we showed that all ST147 clones were XDR and PDR strains harboring various resistance genes in Bangladesh. In addition, the ST147 clone was previously only isolated in the hospital environment, but a strain of ST147 that co-produces NDM-9 and CTX-M-15 has been isolated from wastewater in Switzerland (Nüesch-Inderbinen et al., 2018). This suggests that these PDR strains may already be present in our public health and are increasingly spreading under the surface. Therefore, ST147 will increasingly spread as a One Health infectious disease, and its dissemination may carry higher risks than other international high-risk clones such as ST11, ST15, and ST258.

In recent years, new antibacterial agents against carbapenemase-producing bacteria have been developed one after another and are expected to greatly contribute to the treatment of CPO infection (Ito et al., 2016). However, these new antimicrobials are expensive for some time after approval and are difficult to be readily available in developing countries. As another treatment option for CPO infections, a combination therapy by existing antibiotics is expected. We reported the efficacy of 136 pattern of combination therapies for five types of CPO and non-CPO (Okanda and Matsumoto, 2020). This report mainly suggests that the combined use of protein synthesis inhibitors and cell wall synthesis inhibitor is useful. However, CPOs isolated in South Asia often carry 16SRMTase, and there is concern that combinations using aminoglycosides are ineffective due to their high MIC values for aminoglycosides. Since 91% of CPKP isolates are AMK-high-resistant strains (MIC ≥ 512 μg/ml) harboring 16SRMTase in this study, the effect of combination therapy using aminoglycosides cannot be expected. In our report, it is suggested that the combined use of BPM or IPM with MIN or TGC is effective to AMK-high-resistant strains (Okanda and Matsumoto, 2020). This combination therapy may be effective for some PDR-CPKP isolates in Bangladesh, which are not highly resistant to these antimicrobials. However, as the MIC of the antibiotics used in combination increases, the effect of the combination is limited. Therefore, the increase in PDR strains and in MIC value are particularly serious problems for developing countries.

## Conclusion

Spread of CPKP isolates and PDR strain in Bangladesh warrants the need for the intensive surveillance of AMR and the implementation of an efficient infection control program in developing countries for the management of such infections.

## Data Availability Statement

The original contributions presented in the study are publicly available. This data can be found here: [https://www.ddbj.nig.ac.jp/index.html/ accession numbers: JN676828, KF240808, JN676878, JN676879, AF299299, KP050489, KF055402, KP772120, KP772206, KY418157, EF406115, KU985243, HQ267531, JX442976, AF227505, KJ511462, MK469978, MK412918, MK105834, AY220558, KY402263, JQ808129, CP052141, CP052141, EF682133, GQ438249, KX580704, MK043329, KU603636, KU603660, KY658724, JN108890, and AB759690].

## Author Contributions

TO: conceptualization, data curation, methodology, software, supervision, validation, visualization, writing – original draft, and writing – reviewing and editing. TO and TK: formal analysis. AH, HT, TM, and SN: funding acquisition. TO, AH, AI, and QH: investigation. TO and TM: project administration. All authors contributed to the article and approved the submitted version.

## Conflict of Interest

The authors declare that the research was conducted in the absence of any commercial or financial relationships that could be construed as a potential conflict of interest.

## Footnotes

1 http://blast.ncbi.nlm.nih.gov/Blast.cgi

2 https://eucast.org/clinical_breakpoints/

3 https://bigsdb.pasteur.fr/klebsiella/klebsiella.html
