# Supplementary material for: Characteristics of Carbapenemase-Producing Klebsiella pneumoniae Isolated in the Intensive Care Unit of the Largest Tertiary Hospital in Bangladesh
Source: Front Microbiol. 2021 Jan 15;11:612020. doi: 10.3389/fmicb.2020.612020 (PMC7844882; doi:10.3389/fmicb.2020.612020)
Supplement: Supplementary file 1 [file Table_1.DOCX]

**Supplementary Table S1** Oligoneucleotides used in this study

| Target | Primer name | Sequence (5′-3′) | Reference |
| --- | --- | --- | --- |
| Carbapenemase-encoding genes | | | |
| *bla*_KPC_ | KPC-F  KPC-R | CGTCTAGTTCTGCTGTCTTG  CTTGTCATCCTTGTTAGGCG | Poirel et al., 2011 |
| *bla*_GES_ | GES-F  GES-R | GCTTCATTCACGCACTATT  CGATGCTAGAAACCGCTC | Hong et al., 2012 |
| *bla*_IMP_ | IMP-F  IMP-R | GGAATAGAGTGGCTTAAYTCTC  GGTTTAAYAAAACAACCACC | Poirel et al., 2011 |
| *bla*_VIM_ | VIM-F  VIM-R | GATGGTGTTTGGTCGCATA  CGAATGCGCAGCACCAG | Poirel et al., 2011 |
| *bla*_NDM_ | NDM-F  NDM-R | GGTTTGGCGATCTGGTTTTC  CGGAATGGCTCATCACGATC | Poirel et al., 2011 |
| *bla*_OXA-48-like_ | OXA48-F  OXA48-R | GCGTGGTTAAGGATGAACAC  CATCAAGTTCAACCCAACCG | Poirel et al., 2011 |
| *bla*_BIC_ | BIC-F  BIC-R | TATGCAGCTCCTTTAAGGGC  TCATTGGCGGTGCCGTACAC | Poirel et al., 2011 |
| *bla*_AIM_ | AIM-F  AIM-R | CTGAAGGTGTACGGAAACAC  GTTCGGCCACCTCGAATTG | Poirel et al., 2011 |
| *bla*_GIM_ | GIM-F  GIM-R | TCGACACACCTTGGTCTGAA  AACTTCCAACTTTGCCATGC | Poirel et al., 2011 |
| *bla*_SIM_ | SIM-F  SIM-R | TACAAGGGATTCGGCATCG  TAATGGCCTGTTCCCATGTG | Poirel et al., 2011 |
| *bla*_DIM_ | DIM-F  DIM-R | GCTTGTCTTCGCTTGCTAACG  CGTTCGGCTGGATTGATTTG | Poirel et al., 2011 |
| *bla*_SPM_ | SPM-F  SPM-R | AAAATCTGGGTACGCAAACG  ACATTATCCGCTGGAACAGG | Poirel et al., 2011 |
| Other β-lactamase-encoding genes | | | |
| *bla*_CTX-M-1group_ | CTXM7  CTXM8 | GCG TGA TAC CAC TTC ACC TC  TGA AGT AAG TGA CCA GAA TC | Xu et al., 2005 |
| *bla*_CTX-M-2group_ | CTXM17  CTXM18 | TGA TAC CAC CAC GCC GCT C  TAT TGC ATC AGA AAC CGT GGG | Xu et al., 2005 |
| *bla*_CTX-M-8/25/26group_ | CTXM19  CTXM20 | CAA TCT GAC GTT GGG CAA TG  ATA ACC GTC GGT GAC AAT T | Xu et al., 2005 |
| *bla*_CTX-M-9group_ | CTXM11  CTXM12 | ATC AAG CCT GCC GAT CTG GTT A  GTA AGC TGA CGC AAC GTC TGC | Xu et al., 2005 |
| *bla*_SHV-1/variant_ | SHV_mF  SHV_mR | AGCCGCTTGAGCAAATTAAAC  ATCCCGCAGATAAATCACCAC | Caroline et al., 2010 |
| *bla*_TEM-1/-2/variant_ | TEM-mF  TEM-mR | CATTTCCGTGTCGCCCTTATTC  CGTTCATCCATAGTTGCCTGAC | Caroline et al., 2010 |
| *bla*_OXA-1/variant_ | OXA-1_mF  OXA-1_mR | GGCACCAGATTCAACTTTCAAG  GACCCCAAGTTTCCTGTAAGTG | Caroline et al., 2010 |
| *bla*_MOX-1, MOX-2, CMY-1, CMY-8 to CMY-11_ | MOXMF  MOXMR | GCT GCT CAA GGA GCA CAG GAT  CAC ATT GAC ATA GGT GTG GTG C | P-Perez et al., 2002 |
| *bla*_LAT-1 to LAT-4, CMY-2 to CMY-7, BIL-1_ | CITMF  CITMR | TGG CCA GAA CTG ACA GGC AAA  TTT CTC CTG AAC GTG GCT GGC | P-Perez et al., 2002 |
| *bla* _DHA_ | DHAMF  DHAMR | AAC TTT CAC AGG TGT GCT GGG T  CCG TAC GCA TAC TGG CTT TGC | P-Perez et al., 2002 |
| *bla*_ACC_ | ACCMF  ACCMR | AAC AGC CTC AGC AGC CGG TTA  TTC GCC GCA ATC ATC CCT AGC | P-Perez et al., 2002 |
| *bla*_MIR-1, ACT-1_ | EBCMF  EBCMR | TCG GTA AAG CCG ATG TTG CGG  CTT CCA CTG CGG CTG CCA GTT | P-Perez et al., 2002 |
| *bla* _FOX-1 to FOX-5b_ | FOXMF  FOXMR | AAC ATG GGG TAT CAG GGA GAT G  CAA AGC GCG TAA CCG GAT TGG | P-Perez et al., 2002 |
| Mobilized colistin resistance genes | | | |
| *mcr-1* | Mcr1-mF  Mcr1-mR | AGTCCGTTTGTTCTTGTGGC  AGATCCTTGGTCTCGGCTTG | Rebelo et al., 2018 |
| *mcr-2* | Mcr2-mF  Mcr2-mR | CAAGTGTGTTGGTCGCAGTT  TCTAGCCCGACAAGCATACC | Rebelo et al., 2018 |
| *mcr-3* | Mcr3-mF  Mcr3-mR | AAATAAAAATTGTTCCGCTTATG  AATGGAGATCCCCGTTTTT | Rebelo et al., 2018 |
| *mcr-4* | Mcr4-mF  Mcr4-mR | TCACTTTCATCACTGCGTTG  TTGGTCCATGACTACCAATG | Rebelo et al., 2018 |
| *mcr-5* | Mcr5-mF  Mcr5-mR | ATGCGGTTGTCTGCATTTATC  TCATTGTGGTTGTCCTTTTCTG | Rebelo et al., 2018 |
| *mcr-6* | Mcr6-mF  Mcr6-mR | AGCTATGTCAATCCCGTGAT  ATTGGCTAGGTTGTCAATC | Borowiak et al., 2020 |
| *mcr-7* | Mcr7-mF  Mcr7-mR | GCCCTTCTTTTCGTTGTT  GGTTGGTCTCTTTCTCGT | Borowiak et al., 2020 |
| *mcr-8* | Mcr8-mF  Mcr8-mR | TCAACAATTCTACAAAGCGTG  AATGCTGCGCGAATGAAG | Borowiak et al., 2020 |
| *mcr-9* | Mcr9-mF  Mcr9-mR | TTCCCTTTGTTCTGGTTG  GCAGGTAATAAGTCGGTC | Borowiak et al., 2020 |
| 16S rRNA methylases genes | | | |
| *armA*, | armA-F  armA-R | GGTGCGAAAACAGTCGTAGT  TCCTCAAATATCCTCTATGT | Wangkheimayum et al., 2017 |
| *npmA* | npmA-F  npmA-R | CGGGATCCAAGCACTTTCATACTGACG  CGGAATTCCAATTTTGTTCTTATTAGC | Wangkheimayum et al., 2017 |
| *rmtA* | rmtA-F  rmtA-R | CTAGCGTCCATCCTTTCCTC  TTTGCTTCCATGCCCTTGCC | Wangkheimayum et al., 2017 |
| *rmtB* | rmtB-F  rmtB-R | GGAATTCCATATGAACATCAACGATGCC  CCGCTCGAGTCCATTCTTTTTTATCAAGT | Wangkheimayum et al., 2017 |
| *rmtC* | rmtC-F  rmtC-R | CGAAGAAGTAACAGCCAAAG  GCTAGAGTCAAGCCAGAAAA | Wangkheimayum et al., 2017 |
| *rmtD* | rmtD-F  rmtD-R | TCATTTTCGTTTCAGCAC  AAACATGAGCGAACTGAAGG | Wangkheimayum et al., 2017 |
| *rmtE* | rmtE-F  rmtE-R | ATGAATATTGATGAAATGGTTGC  TGATTGATTTCCTCCGTTTTTG | Hidalgo et al., 2013 |
| *rmtF* | rmtF-F  rmtF-R | GCGATACAGAAAACCGAAGG  ACCAGTCGGCATAGTGCTTT | Hidalgo et al., 2013 |
| *rmtG* | rmtG-F  rmtG-R | AAATACCGCGATGTGTGTCC  ACACGGCATCTGTTTCTTCC | Bueno et al., 2013 |
| Tetracycline resistance genes | | | |
| *tetA* | TetA-mF  TetA-mR | GCTACATCCTGCTTGCCTTC  CATAGATCGCCGTGAAGAGG | Ng et al., 2001 |
| *tetB* | TetB-mF  TetB-mR | TTGGTTAGGGGCAAGTTTTG  GTAATGGGCCAATAACACCG | Ng et al., 2001 |
| *tetC* | TetC-mF  TetC-mR | CTTGAGAGCCTTCAACCCAG  ATGGTCGTCATCTACCTGCC | Ng et al., 2001 |
| *tetD* | TetD-mF  TetD-mR | AAACCATTACGGCATTCTGC  GACCGGATACACCATCCATC | Ng et al., 2001 |
| *tetG* | TetG-mF  TetG-mR | GCTCGGTGGTATCTCTGCTC  AGCAACAGAATCGGGAACAC | Ng et al., 2001 |
| Plasmid-mediated quinolone resistance genes | | | |
| *qnrA* | qnrA-F  qnrA-R | ATTTCTCACGCCAGGATTTG  TGCCAGGCACAGATCTTGAC | Jacoby et al., 2009 |
| *qnrB* | qnrB-F  qnrB-R | CGACCTKAGCGGCACTGAAT  GAGCAACGAYGCCTGGTAGYTG | Jacoby et al., 2009 |
| *qnrS* | qnrS-F  qnrS-R | ACTGCAAGTTCATTGAACAG  GATCTAAACCGTCGAGTTCG | Jacoby et al., 2009 |
| qnrD | qnrD-F  qnrD-R | CGAGATCAATTTACGGGGAATA  AACAAGCTGAAGCGCCTG | Cavaco et al., 2009 |
| Fosfomycin resistance genes | | | |
| *fosA* | FosA-F  FosA-R | ATCTGTGGGTCTGCCTGTCGT  ATGCCCGCATAGGGCTTCT | Liu et al., 2020 |
| *fosA2* | FosA2-F  FosA2-R | GCTGCAATCACTCAACCATC  CACGTGCAGCTCCAGCTT | Liu et al., 2020 |
| *fosA3* | FosA3-F  FosA3-R | GCGTCAAGCCTGGCATTT  GCCGTCAGGGCTGAGAAA | Liu et al., 2020 |
| *fosB* | FosB-F  FosB-R | CAGAGATATTTTAGGGGCTGACA  CTCAATCTATCTTCTAAACTTCCTG | Liu et al., 2020 |
| *fosX* | FosX-F  FosX-R | TGTCCCTCACCTTCGACTCT  TTGCTGGTCTGTGGATTTGC | Liu et al., 2020 |
| Sulphonamide resistance genes | | | |
| *sul1* | sul1-F  sul1-R | TAGCGAGGGCTTTACTAAGC  ATTCAGAATGCCGAACACCG | Hu et al., 2016 |
| *sul2* | sul2-F  sul2-R | CCTGTTTCGTCCGACACAGA  GAAGCGCAGCCGCAATTCAT | Hu et al., 2016 |
| Dihydrofolate reductase encoding genes | | | |
| *dfrA1* | dfrA1-F  dfrA1-R | CTTGTTAACCCTTTTGCCAGA  TTGTGAAACTATCACTAATGGTAG | Hu et al., 2016 |
| *dfrA5* | dfrA5-F  dfrA5-R | ATCGTCGATATATGGAGCGTA  TCCACACATACCCTGGTCCG | Hu et al., 2016 |
| *dfrA12* | dfrA12-F  dfrA12-R | TTAGCCGTTTCGACGCGCAT  ATGAACTCGGAATCAGTACGC | Hu et al., 2016 |
| *dfrA13* | dfrA13-F  dfrA13-R | CTCATCTGCTGGCTATCTCA  GAAACTATCACTAATGGCAGC | Hu et al., 2016 |
| *dfrA17* | dfrA17-F  dfrA17-R | GTTAGCCTTTTTTCCAAATCTGGTATG  TTGAAAATATTATTGATTTCTGCAGTG | Hu et al., 2016 |
| *dfrA27* | dfrA27-F  dfrA27-R | AAGAGTCTGATCGCCCATGCCG  TAAAGCAATAACTTACAATC | Hu et al., 2016 |
| *dfrA14* | dfrA14-F  dfrA14-R | CTGCGAAAGCGAAAAACGGCG  GGAATACTCGGGAAGAAAACA | Brolund et al., 2010 |
| *dfrA24* | dfrA24-F  dfrA24-R | CGTTGCTGCTACTGAGAACG  TGCGGTCTTTCAGAGGACTT | Brolund et al., 2010 |
| *dfrA26* | dfrA26-F  dfrA26-R | GGTAACGCGTCAACAAGGTT  GGCGTGTACTTCGGTGAGAT | Brolund et al., 2010 |

1. Borowiak M, Baumann B, Fischer J, Thomas K, Deneke C, Hammerl JA, et al. (2020). Development of a Novel mcr-6 to mcr-9 Multiplex PCR and Assessment of mcr-1 to mcr-9 Occurrence in Colistin-Resistant Salmonella enterica Isolates From Environment, Feed, Animals and Food (2011-2018) in Germany. Front Microbiol. 11:80.
2. Bueno MF, Francisco GR, O'Hara JA, de Oliveira Garcia D, Doi Y. (2013). Coproduction of 16S rRNA methyltransferase RmtD or RmtG with KPC-2 and CTX-M group extended-spectrum beta-lactamases in Klebsiella pneumoniae. Antimicrob Agents Chemother. 57: 2397–2400.
3. Brolund A, Sundqvist M, Kahlmeter G, Grape M. (2010). Molecular characterisation of trimethoprim resistance in Escherichia coli and Klebsiella pneumoniae during a two year intervention on trimethoprim use. PLoS One. 5(2): e9233.
4. Caroline D., Anaelle D. C., Dominique D., Christine F., Guillaume A. (2010). "Development of a set of multiplex PCR assays for the detection of genes encoding important β-lactamases in Enterobacteriaceae" Journal of Antimicrobial Chemotherapy, Volume 65, Issue 3, March 2010, Pages 490–495,
5. Cavaco LM, Hasman H, Xia S, Aarestrup FM. (2009). qnrD, a novel gene conferring transferable quinolone resistance in Salmonella enterica serovar Kentucky and Bovismorbificans strains of human origin. Antimicrob Agents Chemother. 53: 603–608.
6. Hidalgo L, Hopkins KL, Gutierrez B, Ovejero CM, Shukla S, Douthwaite S, et al. (2013). Association of the novel aminoglycoside resistance determinant RmtF with NDM carbapenemase in Enterobacteriaceae isolated in India and the UK. J Antimicrob Chemother. 68: 1543–1550.
7. Hong, S. S., K. Kim, J. Y. Huh, B. Jung, M. S. Kang S. G. Hong. (2012). "Multiplex PCR for rapid detection of genes encoding class A carbapenemases. Ann Lab Med 2012:32(5) 359-361. https://doi.org/10.3343/alm.2012.32.5.359.
8. Hu LF, Chen GS, Kong QX, Gao LP, Chen X, Ye Y, et al. (2016). Increase in the Prevalence of Resistance Determinants to Trimethoprim/Sulfamethoxazole in Clinical Stenotrophomonas maltophilia Isolates in China. PLoS One. 11(6): e0157693.
9. Jacoby, G. A., N. Gacharna, T. A. Black, G. H. Miller, D. C. Hooper. (2009). Temporal appearance of plasmid-mediated quinolone resistance genes. Antimicrob Agents Chemother 53(4): 1665-1666.
10. Liu P, Chen S, Wu ZY, Qi M, Li XY, Liu CX. (2020). Mechanisms of fosfomycin resistance in clinical isolates of carbapenem-resistant Klebsiella pneumoniae. J Glob Antimicrob Resist. 2020; 22: 238-243.
11. Ng LK, Martin I, Alfa M, Mulvey M. (2001). Multiplex PCR for the detection of tetracycline resistant genes. Mol Cell Probes. 15(4): 209-215.
12. Perez-Perez, F. J. and N. D. Hanson (2002). Detection of plasmid-mediated AmpC beta-lactamase genes in clinical isolates by using multiplex PCR. J Clin Microbiol 40(6): 2153-2162.
13. Poirel L, Walsh TR, Cuvillier V, Nordmann P. (2011). Multiplex PCR for detection of acquired carbapenemase genes. Diagn Microbiol Infect Dis. 70:119–23. https://doi.org/10.1016/j.diagmicrobio.2010.12.002
14. Rebelo AR, Bortolaia V, Kjeldgaard JS, Pedersen SK, Leekitcharoenphon P, Hansen IM, et al. (2018). Multiplex PCR for detection of plasmid-mediated colistin resistance determinants, mcr-1, mcr-2, mcr-3, mcr-4 and mcr-5 for surveillance purposes. Euro Surveill. 23(6): 17-00672.
15. Wangkheimayum, J., D. Paul, D. Dhar, R. Nepram, S. Chetri, D. Bhowmik, et al. (2017). Occurrence of Acquired 16S rRNA Methyltransferase-Mediated Aminoglycoside Resistance in Clinical Isolates of Enterobacteriaceae within a Tertiary Referral Hospital of Northeast India. Antimicrob Agents Chemother 61(6).
16. Xu, L., V. Ensor, S. Gossain, K. Nye and P. Hawkey. (2005). Rapid and simple detection of blaCTX-M genes by multiplex PCR assay. J Med Microbiol 54(Pt 12): 1183-1187.
